# Supplementary material for: Stress and job satisfaction over time, the influence of the managerial position: A bivariate longitudinal modelling of Wittyfit data
Source: PLoS One. 2024 Mar 4;19(3):e0298126. doi: 10.1371/journal.pone.0298126 (PMC10911592; doi:10.1371/journal.pone.0298126)
Supplement: S1 Appendix — (DOCX) [file pone.0298126.s001.docx]

# S1 File. Distribution of outcomes.

Information of distribution of outcomes (stress and job satisfaction) are displayed in S1 Fig 1 and S1 Table 1.

**S1 Fig 1. Distribution of stress and job satisfaction over time.** Bar charts and colored areas plot the distributions of stress and job satisfaction over time.


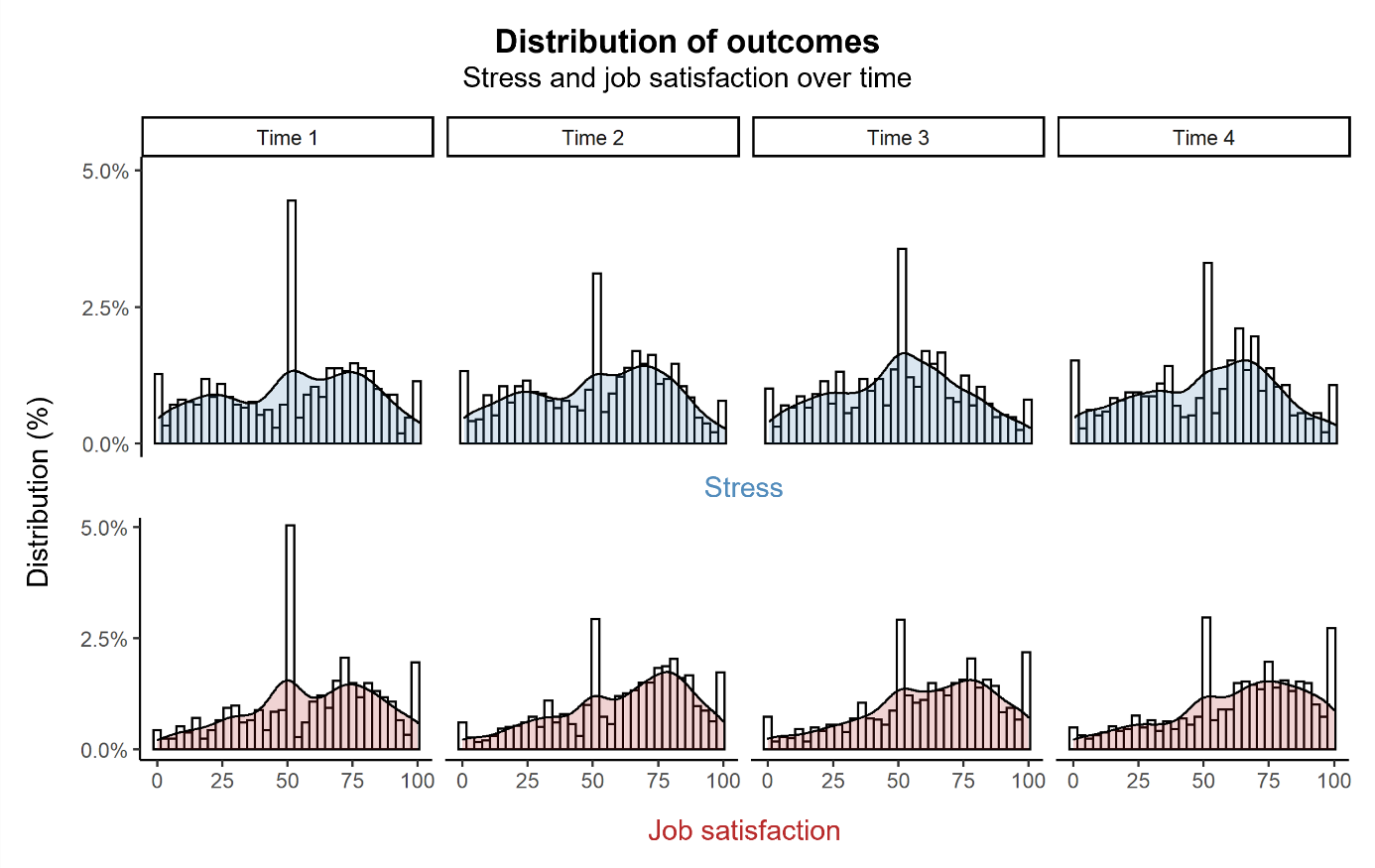


**S1 Table 1. Distribution of records of stress and job satisfaction by job position over time.** The number of individual stress and job satisfaction records each month over the four years is displayed.

| **Year** | **Month** | **Stress** | | | **Job satisfaction** | | | **Total**  **(by month)** | **Total**  **(by year)** |
| --- | --- | --- | --- | --- | --- | --- | --- | --- | --- |
|  |  | **Manager** | **Employee** | **Total** | **Manager** | **Employee** | **Total** |  |  |
| 2018 | Jan. | 3 | 33 | 36 | 3 | 34 | 37 | 73 | 1522 |
|  | Feb. | 1 | 10 | 11 | 1 | 10 | 11 | 22 |  |
|  | March | 12 | 236 | 248 | 12 | 237 | 249 | 497 |  |
|  | April | 8 | 53 | 61 | 8 | 54 | 62 | 123 |  |
|  | May | 0 | 3 | 3 | 0 | 4 | 4 | 7 |  |
|  | June | 1 | 3 | 4 | 1 | 3 | 4 | 8 |  |
|  | July | 4 | 16 | 20 | 4 | 16 | 20 | 40 |  |
|  | Aug. | 3 | 9 | 12 | 3 | 9 | 12 | 24 |  |
|  | Sept. | 2 | 10 | 12 | 2 | 10 | 12 | 24 |  |
|  | Oct. | 46 | 215 | 261 | 47 | 218 | 265 | 526 |  |
|  | Nov. | 9 | 66 | 75 | 9 | 66 | 75 | 150 |  |
|  | Dec. | 3 | 11 | 14 | 3 | 11 | 14 | 28 |  |
| 2019 | Jan. | 0 | 5 | 5 | 0 | 7 | 7 | 12 | 1381 |
|  | Feb. | 5 | 52 | 57 | 4 | 55 | 59 | 116 |  |
|  | March | 3 | 147 | 150 | 4 | 160 | 164 | 314 |  |
|  | April | 1 | 14 | 15 | 1 | 14 | 15 | 30 |  |
|  | May | 0 | 9 | 9 | 0 | 9 | 9 | 18 |  |
|  | June | 40 | 179 | 219 | 41 | 178 | 219 | 438 |  |
|  | July | 0 | 7 | 7 | 0 | 7 | 7 | 14 |  |
|  | Aug. | 0 | 5 | 5 | 0 | 5 | 5 | 10 |  |
|  | Sept. | 15 | 16 | 31 | 15 | 17 | 32 | 63 |  |
|  | Oct. | 7 | 12 | 19 | 7 | 12 | 19 | 38 |  |
|  | Nov. | 17 | 65 | 82 | 17 | 65 | 82 | 164 |  |
|  | Dec. | 12 | 70 | 82 | 12 | 70 | 82 | 164 |  |
| 2020 | Jan. | 11 | 33 | 44 | 11 | 33 | 44 | 88 | 1233 |
|  | Feb. | 8 | 90 | 98 | 8 | 90 | 98 | 196 |  |
|  | March | 36 | 288 | 324 | 36 | 288 | 324 | 648 |  |
|  | April | 0 | 6 | 6 | 0 | 6 | 6 | 12 |  |
|  | May | 0 | 0 | 0 | 0 | 0 | 0 | 0 |  |
|  | June | 3 | 7 | 10 | 3 | 7 | 10 | 20 |  |
|  | July | 0 | 0 | 0 | 0 | 0 | 0 | 0 |  |
|  | Aug. | 0 | 0 | 0 | 0 | 0 | 0 | 0 |  |
|  | Sept. | 1 | 2 | 3 | 1 | 2 | 3 | 6 |  |
|  | Oct. | 2 | 10 | 12 | 2 | 10 | 12 | 24 |  |
|  | Nov. | 6 | 55 | 61 | 6 | 54 | 60 | 121 |  |
|  | Dec. | 9 | 50 | 59 | 9 | 50 | 59 | 118 |  |
| 2021 | Jan. | 54 | 263 | 317 | 54 | 264 | 318 | 635 | 1621 |
|  | Feb. | 11 | 81 | 92 | 11 | 83 | 94 | 186 |  |
|  | March | 7 | 176 | 183 | 8 | 176 | 184 | 367 |  |
|  | April | 3 | 14 | 17 | 4 | 14 | 18 | 35 |  |
|  | May | 6 | 21 | 27 | 5 | 23 | 28 | 55 |  |
|  | June | 1 | 21 | 22 | 1 | 21 | 22 | 44 |  |
|  | July | 6 | 15 | 21 | 2 | 20 | 22 | 43 |  |
|  | Aug. | 0 | 1 | 1 | 0 | 3 | 3 | 4 |  |
|  | Sept. | 3 | 8 | 11 | 3 | 9 | 12 | 23 |  |
|  | Oct. | 5 | 7 | 12 | 9 | 10 | 19 | 31 |  |
|  | Nov. | 2 | 34 | 36 | 5 | 45 | 50 | 86 |  |
|  | Dec. | 3 | 44 | 47 | 7 | 58 | 65 | 112 |  |
