# Supplementary material for: Stress and job satisfaction over time, the influence of the managerial position: A bivariate longitudinal modelling of Wittyfit data
Source: PLoS One. 2024 Mar 4;19(3):e0298126. doi: 10.1371/journal.pone.0298126 (PMC10911592; doi:10.1371/journal.pone.0298126)
Supplement: S2 Appendix — (DOCX) [file pone.0298126.s002.docx]

## S2 File. Statistical Analysis Plan.

**Statistical Analysis Plan**

| Stress and job satisfaction over time, the influence of the managerial position: a bivariate longitudinal modelling of Wittyfit data |
| --- |

Trial registration: Clinicaltrials.gov: NCT02596737.

Date: 14/11/2022

Corresponding author: Rémi Colin

1. Trial summary

1.1. Primary objectives

To assess the simultaneous effect of job position (manager vs. employee) on both stress and job satisfaction in workers using the Wittyfit software. We hypothesize that the managerial position could increase workers’ stress and job satisfaction.

1.2. Secondary objectives

- To explore the influences of sociodemographics, age, seniority, and gender, on stress and job satisfaction by job position
- To evaluate the simultaneous changes in stress and job satisfaction over time (four years), for all workers and by job position, on average and with a group-based method
- To assess the reciprocal influences of stress and job satisfaction over time, for all workers and by job position

1.3. Trial design

Behavioral, self-reported, correlated-clustered, longitudinal, real-world data study from users of the Wittyfit software with two groups defined by worker’s job position, the managers, and the employees.

1.4. Eligibility criteria

1.4.1. Inclusion criteria

- All workers using the Wittyfit software

1.4.2. Exclusion criteria

- Workers with no record of stress or job satisfaction at baseline (year 2018)
- Workers with two or fewer records of stress and job satisfaction over the years (insufficient number of measurements to assess individual changes)
- Workers with no sociodemographics registered
- Workers working in managerless companies

1.5. Primary outcomes measures

The primary outcomes measures are the annual mean levels of workers’ self-reported records in stress and job satisfaction. Both indicators are measured using visual analog scales of Wittyfit.

1.6. Secondary outcome measures

Secondary outcome measures include:

- Job position of the worker, which can be manager or employee
- Worker’s age group (if over or under 40 years old), seniority group (if over or under 5 years), and gender (male or female)
- Company to which the worker belongs
- Time, defined as the year of the measurement, varying from 1 to 4

2. Analysis plan

2.1. Generality

All analyses were performed using R software (version 4.2.1, R Core Team, R Foundation for Statistical Computing, Vienna, Austria), except for group-based multi-trajectory modelling which was performed using Stata (version 17, StataCorp, College Station, USA).

The primary analyses (both cross-sectional and longitudinal) were conducted in the entire study sample. Subgroup longitudinal analyses were performed in both managers and employees to explore the effect of the job position on the evolution of stress and job satisfaction over time.

2.2. Study sample

The study sample consisted of 704 workers using Wittyfit who did not meet the exclusion criteria. Workers were divided in two groups according to their job position, resulting in 81 managers and 623 employees.

2.3. Primary analyses

Workers’ stress and job satisfaction were reported for each group, first across all four years (for cross-sectional analysis) and then year by year (for longitudinal analysis). The primary effect estimates were the mean differences in stress and in job satisfaction, reported with 95% confidence intervals (95CI). Estimates were then turned into Hedge’s g effect size and its 95CI. Stress and job satisfaction were compared between groups using a bivariate random-effect linear model with the company effect as random, (i) cross-sectionally with the workers’ mean levels over all years and (ii) longitudinally by adding the individuals and time effects as random. S2 Fig 1 summarize the analysis process followed throughout the study.

**S2 Fig 1. Study analysis process.** The flowchart shows the order in which the different analyzes were carried out.

2.4. Secondary analyses

Group-based multi-trajectory model was used to identify latent groups of workers, depending on their evolutionary multi-trajectory of stress and job satisfaction. The model selection was based on multiple rules. First, we computed the average posterior probability of assignment (APPA) and the odds of correct classification (OCC) defined by Nagin [28]. Thus, the APPA, which represents the average probability of an individual being assigned to a class given its response pattern, had to be at least 70%, and the OCC, which evaluates across-class separation and thereby indicates how good the latent classes are separated, had to be at least five. Then, we ensured that each trajectory had enough individuals in it. To be retained, each trajectory in the model had to include more than 5% of the total sample (704*5%=35.2, i.e., 36 individuals). Finally, we ensured that the model entropy, which measure the degree of accuracy of the classification of participants into the latent groups defined by the model, was over or equal to 0.80. The model was fitted for several classes ranging from 1 to 10, and the one with the largest number of groups and which complied with the rules was selected. Individuals were then assigned to the group to which they had the highest probability of belonging. Multinomial logistic random-effect model with “company” effect as random was performed to evaluate the effect of the job position on latent bivariate trajectory assignment by comparing the proportion of managers and employees in the groups.

Cross-lagged panel model was applied to explore the putative reciprocal influences of stress and job satisfaction. Three types of effects were measured: (i) autoregressive effects, which measure the stability in constructs over time, (ii) covariance effects, which measure the relationship between variables at the same time, and (iii) cross-lagged effects, which measure the relationship between variables over time. Autoregressive and cross-lagged effects were measured with a lag of one time-point only (e.g., the influence of stress in 2019 on job satisfaction in 2020, or the influence of job satisfaction in 2018 on stress in 2019). Subgroup analysis was conducted to explore the relationships between stress and job satisfaction according to job position.

A total of 17.8% missing values were found, including 18.3% for stress and 17.3% for job satisfaction. Based on the distribution of missing values of the two indicators over the years, we identified that the structure of the missing data was arbitrary and that there was no attrition. As the type of missing values could not be defined with certainty, we considered it as not missing at random. These two assumptions led to conduct sensitivity analyses to ensure the veracity of the results by using multiple datasets, some of which that have been imputed with an imputation method. We then used: (i) an “available-cases” dataset, i.e., namely the original data with the missing values, (ii) a “complete-cases” dataset of 182 individuals (19 managers and 163 employees), two single-imputed datasets, (iii) one imputed with the linear interpolation method, (iv) the other with the “last observation carried forward” method (R software, package “longitudinal data”, command “imputation” with method parameters “linearInterpol” and “locf”) and (v) the “multiple-imputed” dataset, which like the previous dataset has also been imputed but with the multiple imputation method.

Two-level multiple imputation was performed using chained equations with age, seniority, gender, and job position as predictor variables, and individual, time, and company as clustering variables (R software, “mice” package and command). Another alternative, the full case dataset restricted to the period 2019 to 2021, could not be considered due to the inability to compare results from longitudinal models because of a different number of time points.

Unless specified, a p-value <0.05 was considered as statistically significant.
