# Supplementary material for: Stress and job satisfaction over time, the influence of the managerial position: A bivariate longitudinal modelling of Wittyfit data
Source: PLoS One. 2024 Mar 4;19(3):e0298126. doi: 10.1371/journal.pone.0298126 (PMC10911592; doi:10.1371/journal.pone.0298126)
Supplement: S3 Appendix — (DOCX) [file pone.0298126.s003.docx]

# S3 File. Sensitivity analysis results.

Results of sensitivity analysis for all models are displayed from S3 Tables 1 to 7.

**S3 Table 1. Results of sensitivity analysis: univariate mixed models of stress.** Models’ estimates were turned into Hedges’ g effect sizes and 95% confidence intervals. A bold result means that the difference is significant. ‘REF’: reference.

| **Label** | **Available cases** | **Complete cases** | **Linear interpolation** | **Last observation carried forward** | **Multiple imputation** |
| --- | --- | --- | --- | --- | --- |
| Stress by job position (manager REF vs. employee) | 0.13 (–0.10 to 0.36) | 0.09 (–0.38 to 0.58) | 0.11 (–0.11 to 0.36) | 0.10 (–0.13 to 0.34) | 0.14 (–0.09 to 0.38) |
| Managers’ stress by age (<40 REF vs. ≥40) | 0.19 (–0.23 to 0.65) | 0.86 (–0.14 to 1.77) | 0.25 (–0.21 to 0.68) | 0.27 (–0.19 to 0.70) | 0.19 (–0.30 to 0.59) |
| Managers’ stress by seniority (<5 REF vs. ≥5) | –0.06 (–0.48 to 0.39) | –0.06 (–1.16 to 1.04) | –0.08 (–0.48 to 0.39) | –0.06 (–0.48 to 0.39) | –0.06 (–0.47 to 0.40) |
| Managers’ stress by gender (male REF vs. female) | 0.06 (–0.41 to 0.56) | 0.24 (–0.65 to 1.11) | 0.03 (–0.42 to 0.52) | –0.01 (–0.46 to 0.47) | 0.03 (–0.43 to 0.51) |
| Employees’ stress by age (<40 REF vs. ≥40) | –0.01 (–0.18 to 0.14) | 0.17 (–0.15 to 0.47) | –0.02 (–0.18 to 0.14) | –0.01 (–0.16 to 0.16) | 0.00 (–0.16 to 0.16) |
| Employees’ stress by seniority (<5 REF vs. ≥5) | 0.26 (0.10 to 0.43) | 0.46 (0.13 to 0.81) | 0.25 (0.08 to 0.41) | 0.26 (0.08 to 0.41) | 0.28 (0.10 to 0.44) |
| Employees’ stress by gender (male REF vs. female) | 0.02 (–0.14 to 0.17) | 0.05 (–0.27 to 0.36) | 0.01 (–0.14 to 0.16) | 0.01 (–0.14 to 0.16) | 0.04 (–0.12 to 0.19) |

**S3 Table 2. Results of sensitivity analysis: univariate mixed models of job satisfaction.** Models’ estimates were turned into Hedges’ g effect sizes and 95% confidence intervals. A bold result means that the difference is significant. ‘REF’: reference.

| **Label** | **Available cases** | **Complete cases** | **Linear interpolation** | **Last observation carried forward** | **Multiple imputation** |
| --- | --- | --- | --- | --- | --- |
| Job satisfaction by job position (manager REF vs. employee) | –0.65 (–0.89 to –0.42) | –0.68 (–1.13 to –0.19) | –0.63 (–0.86 to –0.39) | –0.62 (–0.85 to –0.38) | –0.70 (–0.94 to –0.47) |
| Managers’ job satisfaction by age (<40 REF vs. ≥40) | –0.31 (–0.76 to 0.13) | –0.06 (–1.02 to 0.81) | –0.35 (–0.82 to 0.07) | –0.34 (–0.82 to 0.07) | –0.30 (–0.76 to 0.12) |
| Managers’ job satisfaction by seniority (<5 REF vs. ≥5) | –0.18 (–0.64 to 0.25) | –0.04 (–1.07 to 1.13) | –0.20 (–0.60 to 0.26) | –0.21 (–0.64 to 0.23) | –0.24 (–0.70 to 0.16) |
| Managers’ job satisfaction by gender (male REF vs. female) | –0.16 (–0.65 to 0.28) | –0.78 (–1.69 to 0.14) | –0.17 (–0.65 to 0.32) | –0.17 (–0.66 to 0.31) | –0.16 (–0.65 to 0.32) |
| Employees’ job satisfaction by age (<40 REF vs. ≥40) | –0.01 (–0.17 to 0.15) | –0.11 (–0.43 to 0.20) | 0.00 (–0.16 to 0.16) | 0.00 (–0.16 to 0.17) | –0.01 (–0.18 to 0.14) |
| Employees’ job satisfaction by seniority (<5 REF vs. ≥5) | –0.33 (–0.50 to –0.16) | –0.41 (–0.77 to –0.09) | –0.32 (–0.50 to –0.17) | –0.33 (–0.50 to –0.16) | –0.37 (–0.54 to –0.20) |
| Employees’ job satisfaction by gender (male REF vs. female) | 0.06 (–0.10 to 0.22) | 0.21 (–0.13 to 0.51) | 0.06 (–0.09 to 0.22) | 0.06 (–0.09 to 0.22) | 0.07 (–0.08 to 0.23) |

**S3 Table 3. Results of sensitivity analysis: bivariate mixed models of stress and job satisfaction.** Models’ estimates were turned into Hedges’ g effect sizes and 95% confidence intervals. A bold result means that the difference is significant. ‘REF’: reference.

| **Label** | **Available cases** | **Complete cases** | **Linear interpolation** | **Last observation carried forward** | **Multiple imputation** |
| --- | --- | --- | --- | --- | --- |
| Stress by job position (manager REF vs. employee) | 0.14 (–0.11 to 0.36) | 0.07 (–0.37 to 0.57) | 0.14 (–0.11 to 0.38) | 0.13 (–0.12 to 0.37) | 0.16 (–0.09 to 0.40) |
| Job satisfaction by job position (manager REF vs. employee) | –0.85 (–1.10 to –0.62) | –0.97 (–1.45 to –0.50) | –0.86 (–1.09 to –0.62) | –0.84 (–1.07 to –0.60) | –0.93 (–1.16 to –0.68) |
| Managers’ stress by age (<40 REF vs. ≥40) | 0.42 (–0.01 to 0.85) | 1.15 (0.22 to 2.05) | 0.48 (0.08 to 0.97) | 0.49 (0.08 to 0.97) | 0.39 (0.02 to 0.90) |
| Managers’ job satisfaction by age (<40 REF vs. ≥40) | –0.44 (–0.89 to –0.01) | –0.16 (–1.05 to 0.84) | –0.49 (–0.89 to 0.00) | –0.48 (–0.88 to 0.01) | –0.42 (–0.82 to 0.07) |
| Managers’ stress by seniority (<5 REF vs. ≥5) | –0.09 (–0.51 to 0.37) | –0.11 (–1.09 to 1.11) | –0.09 (–0.55 to 0.33) | –0.07 (–0.55 to 0.33) | –0.06 (–0.54 to 0.34) |
| Managers’ job satisfaction by seniority (<5 REF vs. ≥5) | –0.22 (–0.65 to 0.23) | –0.06 (–1.17 to 1.02) | –0.26 (–0.66 to 0.22) | –0.27 (–0.69 to 0.20) | –0.30 (–0.70 to 0.18) |
| Managers’ stress by gender (male REF vs. female) | 0.32 (–0.18 to 0.77) | 0.34 (–0.59 to 1.21) | 0.28 (–0.16 to 0.78) | 0.22 (–0.22 to 0.72) | 0.27 (–0.17 to 0.77) |
| Managers’ job satisfaction by gender (male REF vs. female) | –0.25 (–0.73 to 0.23) | –1.06 (–1.95 to –0.16) | –0.28 (–0.73 to 0.21) | –0.29 (–0.73 to 0.20) | –0.26 (–0.70 to 0.23) |
| Employees’ stress by age (<40 REF vs. ≥40) | 0.04 (–0.11 to 0.21) | 0.26 (–0.05 to 0.58) | 0.02 (–0.14 to 0.18) | 0.04 (–0.12 to 0.20) | 0.04 (–0.12 to 0.20) |
| Employees’ job satisfaction by age (<40 REF vs. ≥40) | –0.05 (–0.22 to 0.11) | –0.14 (–0.46 to 0.16) | –0.03 (–0.18 to 0.13) | –0.03 (–0.18 to 0.13) | –0.05 (–0.21 to 0.11) |
| Employees’ stress by seniority (<5 REF vs. ≥5) | 0.34 (0.18 to 0.52) | 0.63 (0.30 to 0.95) | 0.34 (0.19 to 0.52) | 0.35 (0.20 to 0.53) | 0.37 (0.21 to 0.54) |
| Employees’ job satisfaction by seniority (<5 REF vs. ≥5) | –0.47 (–0.63 to –0.30) | –0.53 (–0.92 to –0.24) | –0.46 (–0.63 to –0.29) | –0.46 (–0.63 to –0.29) | –0.50 (–0.68 to –0.34) |
| Employees’ stress by gender (male REF vs. female) | 0.03 (–0.13 to 0.19) | 0.06 (–0.25 to 0.36) | 0.03 (–0.13 to 0.18) | 0.03 (–0.13 to 0.18) | 0.05 (–0.11 to 0.21) |
| Employees’ job satisfaction by gender (male REF vs. female) | 0.05 (–0.11 to 0.22) | 0.27 (–0.03 to 0.59) | 0.04 (–0.12 to 0.19) | 0.04 (–0.12 to 0.19) | 0.05 (–0.12 to 0.20) |

**S3 Table 4. Results of sensitivity analysis: longitudinal mixed model of stress and job satisfaction over time.** Models’ estimates were turned into Hedges’ g effect sizes and 95% confidence intervals. A bold result means that the difference is significant. ‘REF’: reference.

| **Label** | **Available cases** | **Complete cases** | **Linear interpolation** | **Last observation carried forward** | **Multiple imputation** |
| --- | --- | --- | --- | --- | --- |
| Workers’ stress in 2019 (2018 as REF) | 0.02 (–0.09 to 0.13) | 0.06 (–0.15 to 0.27) | 0.00 (–0.11 to 0.10) | 0.02 (–0.09 to 0.12) | 0.02 (–0.09 to 0.12) |
| Workers’ stress in 2020 (2018 as REF) | –0.08 (–0.19 to 0.03) | –0.11 (–0.32 to 0.09) | –0.07 (–0.19 to 0.03) | –0.05 (–0.16 to 0.07) | –0.06 (–0.17 to 0.05) |
| Workers’ stress in 2021 (2018 as REF) | –0.05 (–0.16 to 0.07) | 0.04 (–0.16 to 0.25) | –0.02 (–0.13 to 0.08) | –0.02 (–0.13 to 0.08) | –0.06 (–0.16 to 0.05) |
| Workers’ job satisfaction in 2019 (2018 as REF) | 0.03 (–0.07 to 0.14) | 0.12 (–0.08 to 0.33) | 0.06 (–0.05 to 0.17) | 0.04 (–0.06 to 0.16) | 0.04 (–0.07 to 0.15) |
| Workers’ job satisfaction in 2020 (2018 as REF) | 0.13 (0.02 to 0.24) | 0.23 (0.01 to 0.42) | 0.13 (0.02 to 0.24) | 0.12 (0.01 to 0.22) | 0.14 (0.03 to 0.24) |
| Workers’ job satisfaction in 2021 (2018 as REF) | 0.17 (0.07 to 0.28) | 0.30 (0.11 to 0.53) | 0.16 (0.05 to 0.27) | 0.16 (0.05 to 0.27) | 0.19 (0.08 to 0.30) |
| Managers’ stress in 2019 (2018 as REF) | –0.15 (–0.47 to 0.15) | –0.03 (–0.66 to 0.65) | –0.18 (–0.48 to 0.13) | –0.12 (–0.42 to 0.19) | –0.20 (–0.50 to 0.12) |
| Managers’ stress in 2020 (2018 as REF) | –0.29 (–0.61 to 0.02) | –0.47 (–1.07 to 0.19) | –0.27 (–0.60 to 0.03) | –0.25 (–0.59 to 0.03) | –0.26 (–0.60 to 0.02) |
| Managers’ stress in 2021 (2018 as REF) | –0.31 (–0.63 to –0.01) | 0.13 (–0.46 to 0.79) | –0.21 (–0.54 to 0.07) | –0.21 (–0.54 to 0.08) | –0.29 (–0.62 to 0.00) |
| Managers’ job satisfaction in 2019 (2018 as REF) | 0.06 (–0.26 to 0.37) | 0.15 (–0.50 to 0.79) | 0.15 (–0.16 to 0.45) | 0.12 (–0.20 to 0.41) | 0.13 (–0.19 to 0.43) |
| Managers’ job satisfaction in 2020 (2018 as REF) | 0.10 (–0.19 to 0.42) | 0.01 (–0.56 to 0.67) | 0.11 (–0.22 to 0.40) | 0.09 (–0.24 to 0.38) | 0.11 (–0.21 to 0.41) |
| Managers’ job satisfaction in 2021 (2018 as REF) | 0.34 (0.05 to 0.66) | 0.36 (–0.26 to 1.00) | 0.24 (–0.04 to 0.57) | 0.24 (–0.04 to 0.57) | 0.33 (0.04 to 0.66) |
| Employees’ stress in 2019 (2018 as REF) | 0.04 (–0.07 to 0.16) | 0.08 (–0.14 to 0.29) | 0.03 (–0.09 to 0.14) | 0.04 (–0.08 to 0.16) | 0.05 (–0.07 to 0.16) |
| Employees’ stress in 2020 (2018 as REF) | –0.05 (–0.17 to 0.06) | –0.06 (–0.27 to 0.16) | –0.04 (–0.15 to 0.08) | –0.02 (–0.13 to 0.10) | –0.03 (–0.14 to 0.09) |
| Employees’ stress in 2021 (2018 as REF) | –0.02 (–0.14 to 0.09) | 0.04 (–0.17 to 0.26) | 0.00 (–0.11 to 0.11) | 0.00 (–0.11 to 0.11) | –0.03 (–0.15 to 0.08) |
| Employees’ job satisfaction in 2019 (2018 as REF) | 0.03 (–0.08 to 0.15) | 0.12 (–0.10 to 0.34) | 0.05 (–0.07 to 0.16) | 0.04 (–0.08 to 0.15) | 0.03 (–0.09 to 0.14) |
| Employees’ job satisfaction in 2020 (2018 as REF) | 0.14 (0.04 to 0.27) | 0.24 (0.04 to 0.48) | 0.13 (0.02 to 0.25) | 0.11 (0.00 to 0.23) | 0.13 (0.02 to 0.25) |
| Employees’ job satisfaction in 2021 (2018 as REF) | 0.16 (0.05 to 0.29) | 0.31 (0.09 to 0.53) | 0.14 (0.03 to 0.26) | 0.14 (0.03 to 0.26) | 0.17 (0.06 to 0.28) |
| Stress by job position in 2018 (manager REF vs. employee) | –0.12 (–0.41 to 0.20) | –0.07 (–0.69 to 0.58) | –0.11 (–0.42 to 0.18) | –0.10 (–0.42 to 0.18) | –0.12 (–0.44 to 0.16) |
| Stress by job position in 2019 (manager REF vs. employee) | 0.12 (–0.19 to 0.44) | 0.06 (–0.58 to 0.65) | 0.15 (–0.15 to 0.46) | 0.10 (–0.20 to 0.41) | 0.19 (–0.11 to 0.50) |
| Stress by job position in 2020 (manager REF vs. employee) | 0.19 (–0.13 to 0.51) | 0.44 (–0.22 to 1.07) | 0.18 (–0.13 to 0.48) | 0.19 (–0.12 to 0.49) | 0.18 (–0.15 to 0.47) |
| Stress by job position in 2021 (manager REF vs. employee) | 0.22 (–0.07 to 0.54) | –0.21 (–0.81 to 0.46) | 0.17 (–0.16 to 0.45) | 0.16 (–0.16 to 0.45) | 0.21 (–0.11 to 0.50) |
| Job satisfaction by job position in 2018 (manager REF vs. employee) | –0.68 (–0.99 to –0.38) | –0.83 (–1.44 to –0.22) | –0.65 (–0.98 to –0.36) | –0.65 (–0.97 to –0.35) | –0.69 (–1.01 to –0.39) |
| Job satisfaction by job position in 2019 (manager REF vs. employee) | –0.63 (–0.95 to –0.32) | –0.84 (–1.43 to –0.16) | –0.76 (–1.05 to –0.45) | –0.73 (–1.02 to –0.42) | –0.80 (–1.09 to –0.50) |
| Job satisfaction by job position in 2020 (manager REF vs. employee) | –0.47 (–0.79 to –0.14) | –0.54 (–1.19 to 0.08) | –0.58 (–0.88 to –0.27) | –0.56 (–0.86 to –0.26) | –0.61 (–0.91 to –0.30) |
| Job satisfaction by job position in 2021 (manager REF vs. employee) | –0.74 (–1.05 to –0.41) | –0.86 (–1.52 to –0.22) | –0.69 (–1.01 to –0.38) | –0.68 (–1.00 to –0.37) | –0.78 (–1.10 to –0.47) |

**S3 Table 5. Results of sensitivity analysis: longitudinal mixed models of stress and job satisfaction.** Models’ estimates were turned into Hedges’ g effect sizes and 95% confidence intervals. A bold result means that the difference is significant. ‘REF’: reference.

| **Label** | **Available cases** | **Complete cases** | **Linear interpolation** | **Last observation carried forward** | **Multiple imputation** |
| --- | --- | --- | --- | --- | --- |
| Managers’ stress by age (<40 REF vs. ≥40) | 0.45 (0.02 to 0.88) | 1.21 (0.30 to 2.13) | 0.50 (0.04 to 0.93) | 0.50 (0.04 to 0.93) | 0.41 (–0.05 to 0.83) |
| Managers’ job satisfaction by age (<40 REF vs. ≥40) | –0.44 (–0.90 to –0.02) | –0.15 (–1.14 to 0.69) | –0.49 (–0.93 to –0.04) | –0.49 (–0.91 to –0.02) | –0.43 (–0.92 to –0.03) |
| Managers’ stress by seniority (<5 REF vs. ≥5) | –0.07 (–0.51 to 0.36) | –0.10 (–1.18 to 1.01) | –0.10 (–0.57 to 0.31) | –0.08 (–0.53 to 0.35) | –0.07 (–0.52 to 0.36) |
| Managers’ job satisfaction by seniority (<5 REF vs. ≥5) | –0.24 (–0.69 to 0.19) | –0.07 (–1.31 to 1.01) | –0.27 (–0.70 to 0.18) | –0.28 (–0.70 to 0.17) | –0.31 (–0.74 to 0.14) |
| Managers’ stress by gender (male REF vs. female) | 0.32 (–0.16 to 0.79) | 0.38 (–0.51 to 1.28) | 0.30 (–0.19 to 0.77) | 0.24 (–0.24 to 0.71) | 0.29 (–0.20 to 0.75) |
| Managers’ job satisfaction by gender (male REF vs. female) | –0.24 (–0.71 to 0.23) | –1.05 (–2.03 to –0.15) | –0.27 (–0.76 to 0.19) | –0.28 (–0.77 to 0.18) | –0.25 (–0.74 to 0.20) |
| Employees’ stress by age (<40 REF vs. ≥40) | 0.04 (–0.12 to 0.20) | 0.27 (–0.05 to 0.58) | 0.02 (–0.13 to 0.19) | 0.04 (–0.12 to 0.20) | 0.04 (–0.12 to 0.21) |
| Employees’ job satisfaction by age (<40 REF vs. ≥40) | –0.05 (–0.22 to 0.10) | –0.14 (–0.46 to 0.16) | –0.03 (–0.18 to 0.14) | –0.02 (–0.18 to 0.14) | –0.05 (–0.20 to 0.12) |
| Employees’ stress by seniority (<5 REF vs. ≥5) | 0.36 (0.20 to 0.52) | 0.65 (0.32 to 0.97) | 0.34 (0.18 to 0.51) | 0.36 (0.19 to 0.52) | 0.37 (0.20 to 0.54) |
| Employees’ job satisfaction by seniority (<5 REF vs. ≥5) | –0.46 (–0.62 to –0.28) | –0.56 (–0.90 to –0.22) | –0.46 (–0.62 to –0.29) | –0.46 (–0.62 to –0.29) | –0.50 (–0.67 to –0.34) |
| Employees’ stress by gender (male REF vs. female) | 0.03 (–0.14 to 0.18) | 0.07 (–0.25 to 0.40) | 0.03 (–0.13 to 0.19) | 0.03 (–0.13 to 0.19) | 0.06 (–0.10 to 0.22) |
| Employees’ job satisfaction by gender (male REF vs. female) | 0.05 (–0.11 to 0.21) | 0.28 (–0.03 to 0.59) | 0.05 (–0.11 to 0.21) | 0.04 (–0.11 to 0.21) | 0.05 (–0.10 to 0.22) |

**S3 Table 6. Results of sensitivity analysis: group-based multi-trajectory model metrics and posterior analysis (random-effect multinomial logistic model).** A bold result means that the relationship is significant. ‘APPA’: average posterior probability of assignment, ‘OCC’: odds of correct classification.

| **Label** | **Available cases** | **Complete cases** | **Linear interpolation** | **Last observation carried forward** | **Multiple imputation** |
| --- | --- | --- | --- | --- | --- |
| Optimal number of classes | 3 | 2 | 3 | 3 | 3 |
| Minimum group size/total sample (%) | 102/704 (14.5%) | 75/182 (41.2%) | 129/704 (18.3%) | 133/704 (18.9%) | 119/704 (16.9%) |
| Minimum APPA (%) | 88.5% | 93.1% | 90.3% | 92.1% | 89.2% |
| Minimum OCC | 5.73 | 12.6 | 8.01 | 7.65 | 7.38 |
| Entropy | 0.80 | 0.80 | 0.83 | 0.83 | 0.82 |
| Posterior analysis: influence of job position on multi-trajectory assignment | <0.001 | 0.3 | <0.001 | <0.001 | <0.001 |

**S3 Table 7. Results of sensitivity analysis: cross-lagged panel model (covariances and cross-lagged effects).** Models’ estimates were turned into Hedges’ g effect sizes and 95% confidence intervals. A bold result means that the relationship is significant.

| **Label** | **Available cases** | **Complete cases** | **Linear interpolation** | **Last observation carried forward** | **Multiple imputation** |
| --- | --- | --- | --- | --- | --- |
| Covariance of 2018 stress and job satisfaction in workers | –0.58 (–0.70 to –0.46) | –0.37 (–0.49 to –0.24) | –0.57 (–0.69 to –0.45) | –0.57 (–0.69 to –0.45) | –0.59 (–0.71 to –0.46) |
| Covariance of 2019 stress and job satisfaction in workers | –0.40 (–0.52 to –0.28) | –0.10 (–0.22 to 0.03) | –0.45 (–0.58 to –0.33) | –0.43 (–0.56 to –0.31) | –0.49 (–0.61 to –0.37) |
| Covariance of 2020 stress and job satisfaction in workers | –0.52 (–0.64 to –0.39) | –0.31 (–0.43 to –0.18) | –0.61 (–0.74 to –0.49) | –0.62 (–0.74 to –0.49) | –0.62 (–0.74 to –0.50) |
| Covariance of 2021 stress and job satisfaction in workers | –0.46 (–0.58 to –0.34) | –0.32 (–0.45 to –0.20) | –0.54 (–0.67 to –0.42) | –0.60 (–0.73 to –0.48) | –0.63 (–0.76 to –0.51) |
| Cross-lagged effect of 2018 stress on 2019 job satisfaction in workers | –0.16 (–0.29 to –0.04) | –0.14 (–0.26 to –0.02) | –0.17 (–0.29 to –0.04) | –0.20 (–0.32 to –0.08) | –0.15 (–0.27 to –0.03) |
| Cross-lagged effect of 2019 stress on 2020 job satisfaction in workers | –0.07 (–0.19 to 0.06) | 0.03 (–0.09 to 0.16) | –0.12 (–0.25 to 0.00) | –0.07 (–0.20 to 0.05) | –0.08 (–0.21 to 0.04) |
| Cross-lagged effect of 2020 stress on 2021 job satisfaction in workers | –0.33 (–0.45 to –0.21) | –0.20 (–0.32 to –0.07) | –0.30 (–0.42 to –0.18) | –0.28 (–0.41 to –0.16) | –0.25 (–0.37 to –0.13) |
| Cross-lagged effect of 2018 job satisfaction on 2019 stress in workers | –0.07 (–0.19 to 0.05) | –0.06 (–0.18 to 0.07) | –0.10 (–0.23 to 0.02) | –0.10 (–0.22 to 0.03) | –0.09 (–0.21 to 0.04) |
| Cross-lagged effect of 2019 job satisfaction on 2020 stress in workers | –0.27 (–0.39 to –0.15) | –0.27 (–0.40 to –0.15) | –0.27 (–0.40 to –0.15) | –0.24 (–0.36 to –0.12) | –0.26 (–0.38 to –0.13) |
| Cross-lagged effect of 2020 job satisfaction on 2021 stress in workers | –0.07 (–0.20 to 0.05) | –0.05 (–0.18 to 0.07) | –0.09 (–0.21 to 0.04) | –0.13 (–0.26 to –0.01) | –0.11 (–0.23 to 0.01) |
| Covariance of 2018 stress and job satisfaction in managers | –0.62 (–0.98 to –0.27) | –0.34 (–0.69 to 0.01) | –0.61 (–0.96 to –0.26) | –0.61 (–0.96 to –0.26) | –0.62 (–0.97 to –0.27) |
| Covariance of 2019 stress and job satisfaction in managers | –0.53 (–0.88 to –0.18) | –0.02 (–0.38 to 0.33) | –0.60 (–0.96 to –0.25) | –0.57 (–0.92 to –0.21) | –0.57 (–0.92 to –0.22) |
| Covariance of 2020 stress and job satisfaction in managers | –0.30 (–0.65 to 0.05) | 0.00 (–0.35 to 0.35) | –0.39 (–0.74 to –0.04) | –0.44 (–0.79 to –0.09) | –0.48 (–0.83 to –0.12) |
| Covariance of 2021 stress and job satisfaction in managers | –0.13 (–0.48 to 0.22) | –0.01 (–0.36 to 0.34) | 0.07 (–0.29 to 0.42) | –0.24 (–0.59 to 0.11) | –0.33 (–0.68 to 0.03) |
| Cross-lagged effect of 2018 stress on 2019 job satisfaction in managers | 0.03 (–0.32 to 0.38) | –0.07 (–0.42 to 0.28) | 0.01 (–0.35 to 0.36) | –0.02 (–0.37 to 0.33) | 0.04 (–0.31 to 0.39) |
| Cross-lagged effect of 2019 stress on 2020 job satisfaction in managers | –0.02 (–0.37 to 0.33) | 0.09 (–0.26 to 0.44) | –0.06 (–0.41 to 0.30) | 0.04 (–0.31 to 0.39) | –0.02 (–0.37 to 0.33) |
| Cross-lagged effect of 2020 stress on 2021 job satisfaction in managers | –0.98 (–1.34 to –0.63) | –0.49 (–0.84 to –0.13) | –1.00 (–1.35 to –0.65) | –0.92 (–1.27 to –0.56) | –0.90 (–1.25 to –0.55) |
| Cross-lagged effect of 2018 job satisfaction on 2019 stress in managers | –0.12 (–0.47 to 0.24) | –0.21 (–0.56 to 0.15) | –0.13 (–0.48 to 0.23) | 0.04 (–0.31 to 0.39) | –0.16 (–0.51 to 0.19) |
| Cross-lagged effect of 2019 job satisfaction on 2020 stress in managers | –0.26 (–0.61 to 0.09) | 0.03 (–0.33 to 0.38) | –0.30 (–0.66 to 0.05) | –0.42 (–0.77 to –0.06) | –0.22 (–0.57 to 0.14) |
| Cross-lagged effect of 2020 job satisfaction on 2021 stress in managers | –0.55 (–0.90 to –0.20) | –0.45 (–0.80 to –0.10) | –0.62 (–0.97 to –0.26) | –0.54 (–0.89 to –0.19) | –0.38 (–0.74 to –0.03) |
| Covariance of 2018 stress and job satisfaction in employees | –1.61 (–1.96 to –1.26) | –1.03 (–1.39 to –0.68) | –1.58 (–1.93 to –1.23) | –1.58 (–1.93 to –1.23) | –1.64 (–1.99 to –1.29) |
| Covariance of 2019 stress and job satisfaction in employees | –1.05 (–1.40 to –0.70) | –0.27 (–0.62 to 0.08) | –1.19 (–1.54 to –0.84) | –1.15 (–1.50 to –0.80) | –1.31 (–1.66 to –0.96) |
| Covariance of 2020 stress and job satisfaction in employees | –1.48 (–1.84 to –1.13) | –0.95 (–1.30 to –0.60) | –1.75 (–2.11 to –1.40) | –1.74 (–2.10 to –1.39) | –1.75 (–2.10 to –1.40) |
| Covariance of 2021 stress and job satisfaction in employees | –1.36 (–1.71 to –1.01) | –1.01 (–1.36 to –0.66) | –1.64 (–2.00 to –1.29) | –1.74 (–2.10 to –1.39) | –1.82 (–2.17 to –1.47) |
| Cross-lagged effect 2018 stress on 2019 job satisfaction in employees | –0.54 (–0.89 to –0.19) | –0.43 (–0.78 to –0.08) | –0.55 (–0.90 to –0.20) | –0.64 (–0.99 to –0.29) | –0.51 (–0.86 to –0.16) |
| Cross-lagged effect of 2019 stress on 2020 job satisfaction in employees | –0.21 (–0.56 to 0.14) | 0.07 (–0.28 to 0.42) | –0.36 (–0.71 to –0.01) | –0.24 (–0.59 to 0.11) | –0.25 (–0.60 to 0.10) |
| Cross-lagged effect of 2020 stress on 2021 job satisfaction in employees | –0.75 (–1.10 to –0.40) | –0.41 (–0.76 to –0.06) | –0.66 (–1.01 to –0.31) | –0.63 (–0.98 to –0.28) | –0.54 (–0.89 to –0.19) |
| Cross-lagged effect of 2018 job satisfaction on 2019 stress in employees | –0.14 (–0.49 to 0.21) | –0.11 (–0.46 to 0.24) | –0.23 (–0.58 to 0.12) | –0.26 (–0.61 to 0.09) | –0.16 (–0.52 to 0.19) |
| Cross-lagged effect of 2019 job satisfaction on 2020 stress in employees | –0.72 (–1.07 to –0.37) | –0.79 (–1.14 to –0.44) | –0.73 (–1.08 to –0.38) | –0.60 (–0.95 to –0.25) | –0.71 (–1.06 to –0.36) |
| Cross-lagged effect of 2020 job satisfaction on 2021 stress in employees | –0.06 (–0.41 to 0.29) | –0.01 (–0.36 to 0.34) | –0.10 (–0.46 to 0.25) | –0.28 (–0.63 to 0.08) | –0.21 (–0.56 to 0.14) |
