# Supplementary material for: Stress and job satisfaction over time, the influence of the managerial position: A bivariate longitudinal modelling of Wittyfit data
Source: PLoS One. 2024 Mar 4;19(3):e0298126. doi: 10.1371/journal.pone.0298126 (PMC10911592; doi:10.1371/journal.pone.0298126)
Supplement: Attachment — Submitted filename: Response To Reviewers.docx [file pone.0298126.s006.docx]

*Physiological and Psychosocial Stress*

**–** UMR **CNRS** 6024, LaPSCo

– **UniversitÉ ClERMONT AUVERGNE**

63 000 Clermont-Ferrand, France

[Frederic.Dutheil@uca.fr](mailto:Frederic.Dutheil@uca.fr)

Head of the team “Stress”

*Institute of Occupational Medicine*

– Faculty of Medicine

– **UniversitÉ ClERMONT AUVERGNE**

63 000 Clermont-Ferrand, France

[Frederic.Dutheil@uca.fr](mailto:Frederic.Dutheil@uca.fr)

Head of the Institute

*Occupational & Environmental Health*

– **CENTRE HOSPITALIER UNIVERSITAIrE** (**CHU**)

63 000 Clermont-Ferrand, France

[fdutheil@chu-clermontferrand.fr](mailto:fdutheil@chu-clermontferrand.fr)

Head of the department

Rémi COLIN-CHEVALIER, PhD Student

*Université Clermont Auvergne, CNRS, LaPSCo, Physiological and Psychosocial Stress, CHU Clermont-Ferrand, University Hospital of Clermont-Ferrand, Preventive and Occupational Medicine, Wittyfit, F-63000 Clermont-Ferrand, France*

September 13^th^, 2023

Prakash K.C., Ph.D.

Academic Editor

*PLOS ONE*

Dear editor,

My coauthors and I welcomed the review of our manuscript ID **PONE-D-23-14513**, entitled “**Stress and job satisfaction over time, the influence of the managerial position: a bivariate longitudinal modelling of Wittyfit data**”. We have addressed the comments of the reviewers in a revised manuscript and enclose a point-by-point response.

**Journal Requirements**

When submitting your revision, we need you to address these additional requirements.

1. Please ensure that your manuscript meets PLOS ONE's style requirements, including those for file naming. The PLOS ONE style templates can be found at

<https://journals.plos.org/plosone/s/file?id=wjVg/PLOSOne_formatting_sample_main_body.pdf> and

<https://journals.plos.org/plosone/s/file?id=ba62/PLOSOne_formatting_sample_title_authors_affiliations.pdf>

[REPLY] Thank you for your comment. We appreciate the time you devoted to reading our manuscript and helping us to construct an improved version. Your comments have led to changes that have significantly improved this manuscript.

The following corrections have been made to the manuscript:

- a specific style has been applied to all titles regarding their level
- the titles of figures and tables have been coded, and their captions added immediately afterwards
- the affiliation of RC and FD has been corrected, based on a previous publication by FD in the journal PLOS ONE (<https://journals.plos.org/plosone/article?id=10.1371/journal.pone.0279180>)
- information on the corresponding author has been limited to his e-mail address
- addition information (full and short title) was removed

2. Thank you for stating the following in the Competing Interests section:

“I have read the journal's policy and the authors of this manuscript have the following competing interests:

RC, SD, and TC are part of Wittyfit. Other authors have declared that no competing interests exist (FD is responsible for the scientific accuracy of Wittyfit but is not paid by Cegid; as previously published, Wittyfit is a public private partnership with the CHU of Clermont-Ferrand, France).”

Please confirm that this does not alter your adherence to all PLOS ONE policies on sharing data and materials, by including the following statement: “This does not alter our adherence to PLOS ONE policies on sharing data and materials.” (as detailed online in our guide for authors <http://journals.plos.org/plosone/s/competing-interests>).

If there are restrictions on sharing of data and/or materials, please state these. Please note that we cannot proceed with consideration of your article until this information has been declared.

Please include your updated Competing Interests statement in your cover letter; we will change the online submission form on your behalf.

[REPLY] Thank you for your comment. The data availability statement of the acknowledgments section now reads: “Competing interests: RC, SD, and TC are part of Wittyfit. Other authors have declared that no competing interests exist (FD is responsible for the scientific accuracy of Wittyfit but is not paid by Cegid; as previously published, Wittyfit is a public private partnership with the CHU of Clermont-Ferrand, France). This does not alter our adherence to PLOS ONE policies on sharing data and materials.” The cover letter now reads: “We declare some conflicts of interest associated with this publication, and no significant financial support for this work that could have influence its outcome. This does not alter our adherence to PLOS ONE policies on sharing data and materials. As Corresponding Author, I confirm that the manuscript has been read and approved for submission by all named authors.”

3. Please include your full ethics statement in the ‘Methods’ section of your manuscript file. In your statement, please include the full name of the IRB or ethics committee who approved or waived your study, as well as whether or not you obtained informed written or verbal consent. If consent was waived for your study, please include this information in your statement as well.

[REPLY] Thank you for your comment. The recruitment subsection of the materials and methods section now reads: “This study received approval from both the National Commission for Data Protection and Liberties (CNIL) and the ethics committee of South-East VI (identified on clinicaltrials.gov with the registration number NCT02596737).”

4. We notice that your supplementary files [Appendix S1 to S4] are included in the manuscript file. Please remove them and upload them with the file type 'Supporting Information'. Please ensure that each Supporting Information file has a legend listed in the manuscript after the references list.

[REPLY] Thank you for your comment. All appendixes have been uploaded as separated files and removed from the manuscript.

5. In your Data Availability statement, you have not specified where the minimal data set underlying the results described in your manuscript can be found. PLOS defines a study's minimal data set as the underlying data used to reach the conclusions drawn in the manuscript and any additional data required to replicate the reported study findings in their entirety. All PLOS journals require that the minimal data set be made fully available. For more information about our data policy, please see <http://journals.plos.org/plosone/s/data-availability>.

Upon re-submitting your revised manuscript, please upload your study’s minimal underlying data set as either Supporting Information files or to a stable, public repository and include the relevant URLs, DOIs, or accession numbers within your revised cover letter. For a list of acceptable repositories, please see <http://journals.plos.org/plosone/s/data-availability#loc-recommended-repositories>. Any potentially identifying patient information must be fully anonymized.

Important: If there are ethical or legal restrictions to sharing your data publicly, please explain these restrictions in detail. Please see our guidelines for more information on what we consider unacceptable restrictions to publicly sharing data: <http://journals.plos.org/plosone/s/data-availability#loc-unacceptable-data-access-restrictions>. Note that it is not acceptable for the authors to be the sole named individuals responsible for ensuring data access.

We will update your Data Availability statement to reflect the information you provide in your cover letter.

[REPLY] Thank you for your comment. Datasets are full available as supplementary materials, both for original and imputed datasets.

**Additional Editor Comments**

Psychological wellbeing at work is an important aspect of working life connected to overall wellbeing and health of an individual. Being topical it has gained a lot of research attention in recent decades. Work-related psychosocial demands are highlighted as key aspects to employee wellbeing in existing literatures. This is a well written manuscript, which could be a good addition to the current body of scientific literatures and comes up with important dimension that is rarely examined and I am impressed by the analyses.

[REPLY] Thank you for your very positive comment.

However, there are few important aspects missing in the manuscript, which are nicely summarized by Reviewer 1. Given the importance of this research, I would like to give the authors a chance of revision. I strongly recommend major revisions based on the comments given by reviewer 1.

[REPLY] Thank you for your comment. As you will see below, we have addressed all the editor’ and reviewers’ comments and made additions and modifications to our manuscript and cover letter accordingly. We hope that the changes made will have met your expectations.

**Review Comments to the Author:**

**Reviewer 1**

In this article, the authors have explored the bivariate effect of managerial position on stress and job satisfaction of the employees and the inter-relationship of these indicators over time. The influence of age, seniority, and gender on stress and job satisfaction was also assessed. This is an important topic as stress is becoming increasingly common in the everyday life of the modern workforce and might have a negative impact on job satisfaction. Increased job stress and low job satisfaction not only impact the individual’s overall well-being but also impacts the work organization and families. The scientific writing is good, the statistical analysis is nicely done, and the results are presented well.

[REPLY] Thank you for your very positive comment.

However, the are some major concerns:

The sample size presented in the study is quite small and belongs to three companies. How are the companies selected? What sector of work do these companies belong to? It is well documented that the level of job stress and job satisfaction varies by the work sector for both employees and managers. Hence, it could not be generalized to whole working population without having sufficient background information on the work sector.

[REPLY] Thank you for your comment. We appreciate the time you devoted to reading our manuscript and helping us to construct an improved version. Your comments have led to changes that have significantly improved this manuscript.

Companies were selected according to two criteria: (1) they were already using the Wittyfit platform in the first year of the study (2018), and (2) they had to include managers and employees among their workers who used Wittyfit. For ease of understanding, the following statement have been added to the inclusion/exclusion criteria subsection: “All companies that were not yet Wittyfit customers in 2018 have been excluded, as well as those with no managers, as it was impossible to measure the effect of job position in these companies.”

Wittyfit is a solution deployed by numerous French companies in a wide variety of sectors and is therefore not targeted at any particular industry. As mentioned in the recruitment subsection: Wittyfit is a digital web software designed by Cegid (Lyon, France) in partnership with the university hospital of Clermont-Ferrand, implemented in numerous French companies from any industry sector [Dutheil, 2017]”. In particular, the three companies included in the study come from three different sectors. As this information could be used to identify them, we have chosen to conceal it. However, for ease of understanding, the following statement have been added to the participant subsection: “Workers included 81 managers and 623 employees from three different companies with 143, 259 and 302 workers (Fig 1). The three companies belonged to commercial activities and services sector.”

The limitations section now reads: “As it is well documented that the level of job stress and job satisfaction varies by the work sector for both employees and managers [Brown, 2001], our results could not be generalized to whole working population. The low sample size also precluded generalizability. However, the database grows over the years [Dutheil, 2017], and further studies with larger populations and longer time periods could be designed to confirm or add to these initial results.”

Dutheil F, Duclos M, Naughton G, Dewavrin S, Cornet T, Huguet P, et al. Wittyfit-live your work differently: study protocol for a workplace-delivered health promotion. JMIR Research Protocols. 2017;6(4):e6267.

Brown A, Kitchell M, O’Neill T, Lockliear J, Vosler A, Kubek D, et al. Identifying meaning and perceived level of satisfaction within the context of work. Work. 2001;16(3):219–26.

What kind of questionnaires were included in the measurement of job satisfaction and job stress? Or is it just the two components “stress” and “job satisfaction” and a visual analog scale from 0-100 to report the perceived levels? The job stress for example is a broad concept and determined based on several components such as job position, age, seniority, and gender, for example, workplace conditions, kind of work (regular or irregular), workload, etc.

[REPLY] Thank you for your comment. The two components “stress” and “job satisfaction” are effectively two visual analog scales that users can update whenever they wish. Once these two levels have been evaluated, and only then, so that users’ self-measurement is not altered by external factors, users can fill in the corresponding questionnaires. The “stress” questionnaire includes two further items, “workload” and “autonomy”. The “job satisfaction” questionnaire includes six other items, “atmosphere”, “recognition”, “work-life balance”, “meaning of work”, “organization” and “professional values”. Thus, it is “stress” and “job satisfaction” that are assessed, rather than more specific ones such as job stress. To avoid any confusion for readers, we have still revised this section by deleting the reference to questionnaires. It now reads: “During the data collection period, users of the Wittyfit software could assess for the first time or update their stress and job satisfaction levels using the relative analog scales, as often as they wished [Lesage, 2012; Dutheil, 2013; Dutheil, 2017].”

Lesage FX, Berjot S, Deschamps F. Clinical stress assessment using a visual analogue scale. Occup Med (Lond). 2012;62(8):600–5.

Dutheil F, Trousselard M, Perrier C, Lac G, Chamoux A, Duclos M, et al. Urinary interleukin-8 is a biomarker of stress in emergency physicians, especially with advancing age--the JOBSTRESS* randomized trial. PLoS One. 2013;8(8):e71658.

Dutheil F, Pereira B, Moustafa F, Naughton G, Lesage FX, Lambert C. At-risk and intervention thresholds of occupational stress using a visual analogue scale. PLoS One. 2017;12(6):e0178948.

The study does not include the basic characteristics of participants such as socio-economic conditions such as income and education which is an important factor for job stress and job satisfaction. Moreover, without information on educational background and the sector of work, it could be expected that the employees with lower levels of education and the ones who are not comfortable using the software to answer the questions would systematically be excluded from the study.

[REPLY] Thank you for your comment. The limitations now read: “The study suffered from a lack of information on workers’ socio-demographic characteristics, such as employment, level of education or income. It cannot be ruled out that workers with a low level of education or limited computer skills may have been excluded.”

The reduced level of job stress is associated with the perceived psychological well-being/ general well-being of a person and ultimately impacts the level of job satisfaction. There is not any measurement of the general perceived well-being of the participants included in the study. Since the Wittyfit software is designed to evaluate overall health, the health and well-being-related information would be an important covariate/ indicator in this study.

[REPLY] Thank you for your comment. We totally agree with your comment, however we believe that this question belongs in a separated article. In fact, we have addressed this issue in an article entitled “Psychosocial Well-Being Index and Sick Leave in the Workplace: a Structural Equation Modelling of Wittyfit Data”, the preprint of which can be found here: <https://preprints.jmir.org/preprint/48477>. In this article, we set out to create an index of psychosocial well-being using a large set of measurement variables, including stress and job satisfaction. In the present article, since only two of the ten dimensions of psychosocial well-being were available, we have not included it. The limitations now read: “Although stress and job satisfaction are known to be associated with well-being [Locker, 1996] and may therefore appear as an important covariate, we did not measure it.”

Locker D. Work stress, job satisfaction and emotional well-being among Canadian dental assistants. Community Dent Oral Epidemiol. 1996;24(2):133–7.

I would recommend addressing these major issues in the manuscript in order for the manuscript for it to have a better scientific impact and suitable for publication in the journal.

**Reviewer 2**

The manuscript is well written. In the discussion component while highlighting the limitations of the study, please kindly justify how this original research adds to existing knowledge base considering the already existing evidence in this area of research. The limitations of the study need to be clearly highlighted with few justifications. The recent published literature which can be considered for a comparative analysis (although it is not exactly what you covered in the research but has overall general theme of relevance) include:

- Dutheil F, Duclos M, Naughton G, Dewavrin S, Cornet T, Huguet P, Chatard JC, Pereira B. WittyFit—Live Your Work Differently: Study Protocol for a Workplace-Delivered Health Promotion. JMIR Research Protocols. 2017 Apr 13;6(4):e6267.
- Colin-Chevalier R, Dutheil F, Cambier S, Dewavrin S, Cornet T, Baker JS, Pereira B. Methodological issues in analyzing real-world longitudinal occupational health data: a useful guide to approaching the topic. International Journal of Environmental Research and Public Health. 2022 Jun 8;19(12):7023.
- Vialatte L, Pereira B, Guillin A, Miallaret S, Baker JS, Colin-Chevalier R, Yao-Lafourcade AF, Azzaoui N, Clinchamps M, Bouillon-Minois JB, Dutheil F. Mathematical modeling of the evolution of Absenteeism in a University hospital over 12 years. International Journal of Environmental Research and Public Health. 2022 Jul 6;19(14):8236.

[REPLY] Thank you for your comment. We appreciate the time you devoted to reading our manuscript and helping us to construct an improved version. Your comments have led to changes that have significantly improved this manuscript. This study is based on a real data set, not on a data set derived from a study. The results reported in this article therefore constitute real-world evidence. We truly believe that this study could benefit from a comparison with a randomized clinical trial, as is often the case for this type of study. As suggested, we have used the recent literature to complete the limitations of the article. The limitation now reads: “[…] the (Wittyfit) database grows over the years [Dutheil, 2017], and further studies with larger populations and longer time periods could be designed to confirm or add to these initial results. It would also be very valuable to compare the results of this study, based on a real-world database and therefore constitutes real evidence, with those of a randomized clinical trial [Colin-Chevalier, 2022].”

We hope our work will be considered favorably and look forward to hearing from you.

Sincerely yours,

Rémi COLIN
